# A systematic scoping review on the evidence behind debriefing practices for the wellbeing/emotional outcomes of healthcare workers

**DOI:** 10.3389/fpsyt.2023.1078797

**Published:** 2023-03-24

**Authors:** Thomas Rhys Evans, Calvin Burns, Ryan Essex, Gina Finnerty, Ella Hatton, Andrew James Clements, Genevieve Breau, Francis Quinn, Helen Elliott, Lorraine D. Smith, Barry Matthews, Kath Jennings, Jodie Crossman, Gareth Williams, Denise Miller, Benjamin Harold, Philip Gurnett, Lee Jagodzinski, Julie Smith, Wendy Milligan, Marianne Markowski, Peter Collins, Yuki Yoshimatsu, Jordi Margalef Turull, Mark Colpus, Mark L. Dayson, Sharon Weldon

**Affiliations:** ^1^School of Human Sciences, University of Greenwich, London, United Kingdom; ^2^Institute for Lifecourse Development, University of Greenwich, London, United Kingdom; ^3^School of Health Sciences, University of Greenwich, London, United Kingdom; ^4^School of Psychology, Arden University, Coventry, United Kingdom; ^5^Aston Business School, Birmingham, United Kingdom; ^6^School of Applied Social Studies, Robert Gordon University, Aberdeen, United Kingdom; ^7^School of Education, University of Greenwich, London, United Kingdom; ^8^School of Sport and Health Sciences, University of Brighton, Brighton, United Kingdom; ^9^Greenwich Learning and Simulation Centre, University of Greenwich, London, United Kingdom; ^10^Lewisham and Greenwich NHS Trust, London, United Kingdom; ^11^University Hospitals Sussex NHS Foundation Trust, Brighton, United Kingdom

**Keywords:** voice, healthcare, debriefing, emotion, wellbeing, systematic review

## Abstract

**Introduction:**

Debriefings give healthcare workers voice through the opportunity to discuss unanticipated or difficult events and recommend changes. The typical goal of routine debriefings has been to improve clinical outcomes by learning through discussion and reflection of events and then transferring that learning into clinical practice. However, little research has investigated the effects of debriefings on the emotional experiences and well-being of healthcare workers. There is some evidence that debriefings are a multi-faceted and cost-effective intervention for minimising negative health outcomes, but their use is inconsistent and they are infrequently adopted with the specific intention of giving healthcare workers a voice. The purpose of this systematic scoping review is therefore to assess the scope of existing evidence on debriefing practices for the well-being and emotional outcomes of healthcare workers.

**Methods:**

Following screening, 184 papers were synthesised through keyword mapping and exploratory trend identification.

**Results:**

The body of evidence reviewed were clustered geographically, but diverse on many other criteria of interest including the types of evidence produced, debriefing models and practices, and outcomes captured.

**Discussion:**

The current review provides a clear map of our existing understanding and highlights the need for more systematic, collaborative and rigorous bodies of evidence to determine the potential of debriefing to support the emotional outcomes of those working within healthcare.

**Systematic Review Registration:**

https://osf.io/za6rj.

## Introduction

Employee voice is informal and discretionary communication by an employee, about work-related issues with the intent of bringing about improvement or change, to people who might be able to take appropriate action (1). In healthcare, debriefings (e.g. end of shift huddles) can give employees voice through discussion of unanticipated or difficult events, including patient deaths, and the opportunity to recommend changes. The main goal of debriefings has been to improve clinical outcomes by learning through discussion and reflection of events and then transferring that learning into clinical practice (2). Less research attention has focused upon the effects of debriefings on the emotional experiences and wellbeing of healthcare workers (3). Research on this topic is crucial and could facilitate development on the third Global Goal surrounding good health and wellbeing (4). In the UK alone, vacancies for healthcare workers are higher than they have ever been, exacerbated by high numbers of resignations and reports of negative emotional experiences including a lack of belonging and sickness due to stress (5). The purpose of this article is to map the existing literature exploring the impacts of debriefing on the emotional wellbeing of healthcare workers.

### Purpose and forms of debriefing

The idea of speaking up for safety as an extra-role behavior, like in debriefings, is not a new one. Hofmann et al. (6) conceptualized safety citizenship behaviors as comprising six dimensions including voice, whistleblowing and initiating safety-related changes. The structure of these dimensions have been found to be invariant across European national sub-samples (7). In safety-critical industrial sectors, the focus on speaking up for safety has been on improving safety outcomes. In healthcare, the focus of debriefings has typically been upon their impact on patient outcomes by technical learning, team cohesion and affective coping (8). Debriefing typically occurs within teams, and consists of discussion and interpretation of events in the workplace to facilitate learning (9), but research suggests that they may also help healthcare workers with emotional outcomes like coping with patient death [e.g. (10)] and overcoming moral distress experienced when they act against their own ethical values [e.g. (11)]. The need for greater voice and effective emotional support structures, like debriefings, to help build and maintain a resilient workforce has been highlighted in many countries by the challenges faced by healthcare workers during the COVID-19 pandemic (12).

There has been wide proliferation of a number of different models and approaches to debriefing, with previous reviews reporting a diverse range of purposes, methodological variations and outcomes [e.g. (13, 14)]. The existing literature tends to be grouped into either (a) guided team discussions to support post-event learning or (b) structured clinical debriefing strategies designed to minimize the psychological consequences of traumatic events (9). Both have the potential to influence the wellbeing and affective experiences of healthcare workers.

First, models focused upon learning include the REFLECT [Review the event, Encourage team participation, Focused feedback, Listen to each other, Emphasize key points, Communicate clearly, Transform the future; (15)], a scripted debriefing tool, TALK [Target, Analysis, Learning, Key Actions; (16)], a framework for supportive dialog which can be facilitated by any individual within a team (16) and PEARLS [The Promoting Excellence and Reflective Learning in Simulation; (17)], a tool with scripted language to guide debriefing. These learning models of debriefing typically evaluate performance and patient outcomes and have very rarely considered the potential emotional consequences (e.g. providing potential to digest daily stressors).

Second, models focused upon minimizing post-traumatic stress and other such mental health outcomes like depression include Critical Incident Debriefing (linked with Critical Incident Stress Management; CISM), Psychological Debriefing and Trauma Risk Management (TRiM). These models can encapsulate quite a range of debriefing practices (18), and can include discussions of the traumatic event, personal meanings attributed to the event, emotional responses, as well as psychoeducation concerning coping responses (19).

Research to date has suggested limited efficacy of the use of CISM as a debriefing intervention for the emotional responses of workers. Tuckey and Scott (19) found that there was no statistical difference in post-traumatic stress or psychological distress scores between fire-fighters accessing CISM, a stress management intervention and a non-active control group. Existing reviews have also been critical of the use of CISM following exposure to trauma. Rose et al. (20) concluded that single-session CISM are not efficacious in reducing the adverse impact of traumatic events in primary victims of trauma. Furthermore, Roberts et al. (21) concluded that there is a weak evidence base for the use of psychological interventions, including CISM, following traumatic events. Magyar and Theophilos (22), however, argued that such results need to be interpreted cautiously as CISM was designed for groups that have shared exposure to a potentially traumatic incident.

Psychological Debriefing has been the subject of contentious debate, particularly surrounding the potential to cause harm to those engaging with it (23). This was summarized by a Cochrane review, which concluded a lack of value of psychological debriefing in preventing PTSD, with two trials finding that this form of debriefing worsened symptoms (20). Debates are ongoing as to whether for the intended audience (emergency personnel and first responders), this strategy may yet still prove beneficial for some dimensions of psychological wellbeing [e.g. (24)]. A key barrier to such knowledge is the heterogeneous methodological quality in evaluation of practices (14).

TriM consists of peer support for those that have experienced a traumatic event. The role of debriefing has been suggested to be favorable compared to CISM approaches, as it does not require participants to talk through details of the event (25). Furthermore, there is a growing support for its application in hierarchical organizations (26), and healthcare specifically [e.g. (27)]. However, Palmer et al. (25) note the limited robust evidence supporting this approach.

While there have been many diverse bodies of evidence exploring these various practices, there have been a number of concerns raised. For example, some debriefing practices, like providing single sessions without follow-up, are concluded to be detrimental to healthcare workers’ wellbeing (28). Consistency in practice has also been discussed in that staff are seldom prepared for engaging with, or delivering, these practices (29). Quality of evidence is a critique regularly raised, and such is the quality and diversity of works represented by this body of literature, many reviews have concluded that no firm recommendations for practice can be made [e.g. (13)].

### The current review

Debriefing holds the potential to represent an accessible, flexible and low-cost intervention for a community of workers in desperate need for emotional support to complete demanding but vital work (5). Given the diversity in available practices, clearer guidance for how to adopt debriefing is vital, and such guidance should be directly informed by clear consensuses and mature bodies of evidence. It is of crucial importance to identify and understand the various types of debriefing adopted in contemporary practice, and to understand their efficacy for giving healthcare workers a voice to improve their emotional experiences and wellbeing. A comprehensive review of this work has not yet been completed, demonstrating the need for a systematic mapping of the existing literature to inform future priorities, directions and practice recommendations.

The aim of this article is to map existing evidence on debriefing practices for the wellbeing and emotional outcomes of healthcare workers. We investigate: “How have the effects of debriefing practices on the wellbeing/emotional outcomes of healthcare workers been studied to-date?.” The current article represents a systematic scoping review to explore the state of current understanding with respect to diversity and efficacy of practices. In doing so, we aim to promote developments in understanding and practice for the debrief interventions available to improve the emotional wellbeing of healthcare staff (following both traumatic incidents and day-to-day practices) and to provide greater employee voice in healthcare.

## Methods

### Preregistration

This project was preregistered on the Open Science Framework, November 22nd 2021^1^ using the Inclusive Systematic Review Preregistration Form (30). A number of deviations from the preregistration were necessary for mostly practical reasons, although they are unlikely to have influenced any of the major conclusions of the project. These deviations have been detailed alongside their justification here.^2^

### Search strategy

Our full search criteria can be found in Appendix 1 and was applied to the Scopus and EBSCO (Medline, PsycInfo, and CINAHL) databases on 24 November 2021. 5,182 works were identified through this initial search. The review was exploratory in nature, and we had no clear expectations of the sources and type of evidence we would encounter and thus did not exclude on the basis of the study design. Plans to source grey literature and file-drawer manuscripts were considered excessive due to the high number of sources identified through this initial search, and difficulty in identifying credible sources and the subsequent lack of rigorous sources identified.

### Screening method

The online screening platform, Rayyan (31), was adopted to facilitate an initial title/abstract screening. As driven by PICO (population, intervention, control and outcomes), studies had to conform to all three of the following to be included:

1. A central practice focus must be on debriefing, regardless of whether it is reported as an intervention, response to specific event, or daily practice. These can sometimes be referred to as briefings or huddles and are inclusive of day-to-day practices and one-off trauma-based interventions.
2. A central sample or focus should be upon healthcare staff and/or healthcare profession. These should be interpreted broadly.
3. The work should include a note of affective/emotional outcomes of debriefing. These should be interpreted broadly and can include mood, specific emotions and emotional states, coping, resilience, psychological health outcomes, wellbeing ratings and similar.

The title and abstract of each paper was assessed by RE (*n* = 1,040) or TRE (*n* = 4,147) to assess compliance with all three criteria. During this process, 193 papers were coded as ‘unsure’ and subsequently assessed by the core research team (RE, TRE, CB, and GF) who met online to discuss each case. From this, 151 were collaboratively agreed to be relevant when consistently erring on the side of inclusion. In total, 271 papers were considered to meet the three criteria.

### Data extraction

All 271 papers were then subject to a full-text review by two members of the wider research team (see ‘Investigation’ in Appendix 2). Twenty-one researchers were recruited from an open call for volunteers circulated by the Institute for Lifecourse Development at the University of Greenwich, or through personal contacts of the study leads. All researchers involved in the review process were provided a 40-min training session^3^ which featured instructions on the review process and opportunities to practice coding using a set of example works. Each researcher attended the training live or viewed the recording post-training and independently self-selected and coded between 5 and 37 papers (*M* = 24). At all review stages, raters were offered the opportunity to exclude themselves from rating the specific article if they had a conflict of interest or other such issue. All data were collected through Google Forms and all forms and data can be found on the OSF project page.^4^

**Figure 1:**
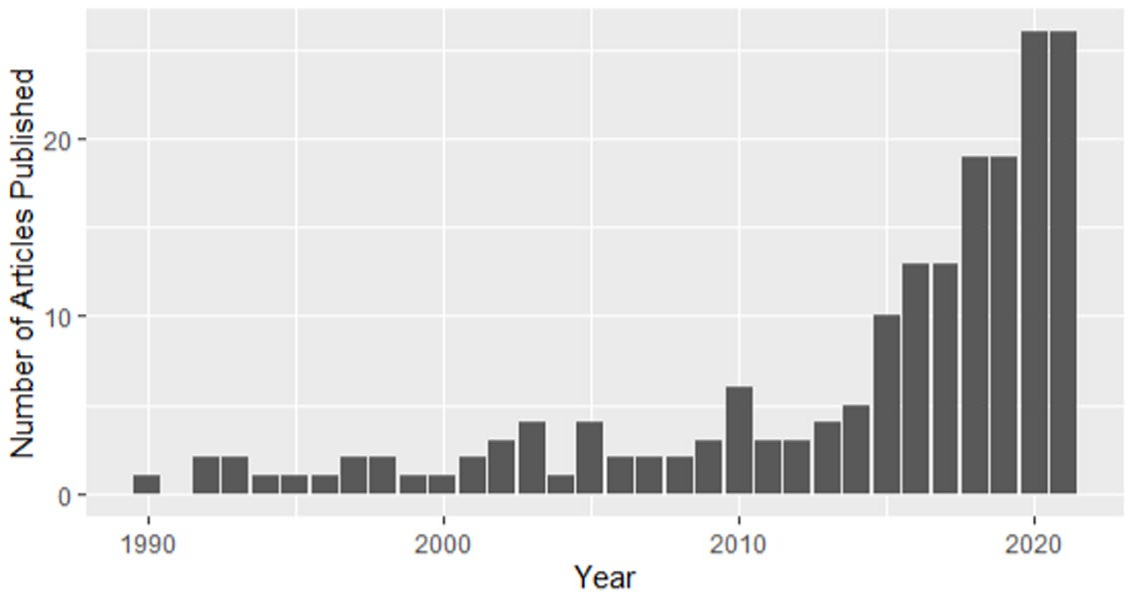
Articles published by date of publication.

Where texts were unavailable through university licences, the project lead attempted to procure a copy through Google Scholar, ResearchGate, Academia.edu, OSF and other such scientific hubs/libraries. Where available, copies were uploaded to a shared private OSF page for accessibility. Following this process, 34 works were excluded on the basis of language (*n* = 4) and full-text accessibility (*n* = 30). Of the 30, 19 were inaccessible journal articles, 3 were book chapters and 8 were doctoral theses. The majority of these (18/30) were published before 2015, when research attention in the field increased (see Figure 1). For each remaining paper, reviewers initially confirmed whether the work met the original 3 criteria, and then coded the following nine characteristics:

1. Country of Study (country where data collection occurred)
2. Type of study (Empirical/Case Study/Theoretical/Review)
3. Sample size (Total N)
4. Population type/specialism (e.g. Paramedics)
5. Regularity of event (e.g. trauma or routine)
6. Whether real-world or simulation setting
7. Specific wellbeing outcome (e.g. PANAS score)
8. Type of Debriefing model (e.g. none, Critical Incident Stress Management)
9. Support provided (e.g. whether facilitated by external party)

To provide an indication of inter-rater agreement, two of the nine criteria were evaluated for similarity between coders. As many of the criteria were recorded as open responses, we present agreement on the forced-choice ‘type of study’ and ‘regularity of event’ responses. These were chosen on the basis of qualitative feedback from coders as to the easiest and most complex coding decisions, respectively, thereby providing an indicative range of agreement for the study. Where more than 2 coders evaluated a paper, we compared agreement between the first two chronologically. For both questions, the coders could provide a free-entry ‘other’ response and as such these were interpreted by TRE on a case-by-case basis as similar (e.g. “Editorial/Opinion” with “Letter”) or contradictory (“Specific Incident” with “Routine”). Of 238 judgement pairs, there were 23 disagreements (~10%) on study design, most commonly due to the ‘Case Study’ category overlapping with other options (e.g. empirical and opinion), and 48 disagreements (~20%) on regularity where most were direct contradictions.

For each paper, the two independent ratings of each manuscript were shared with a member of the core research team who reviewed the responses alongside the original manuscript. They were then asked to provide the most accurate judgement possible based upon the information they had. They were free to decide in favor of one review, to combine responses, or to make a different judgement as they saw fit. Each team member recorded between 42 and 69 manuscripts (*M* = 59). The analyses reported in this manuscript use this final third review as the primary data source.

Of the 237 papers, 53 were excluded upon this third review, most commonly due to not meeting the debriefing focus criteria (*n* = 24). A healthcare population (*n* = 16) and emotion outcome (*n* = 18) were also absent in a number of manuscripts. Five manuscripts were excluded on the basis of more than one criteria.

**Figure 2:**
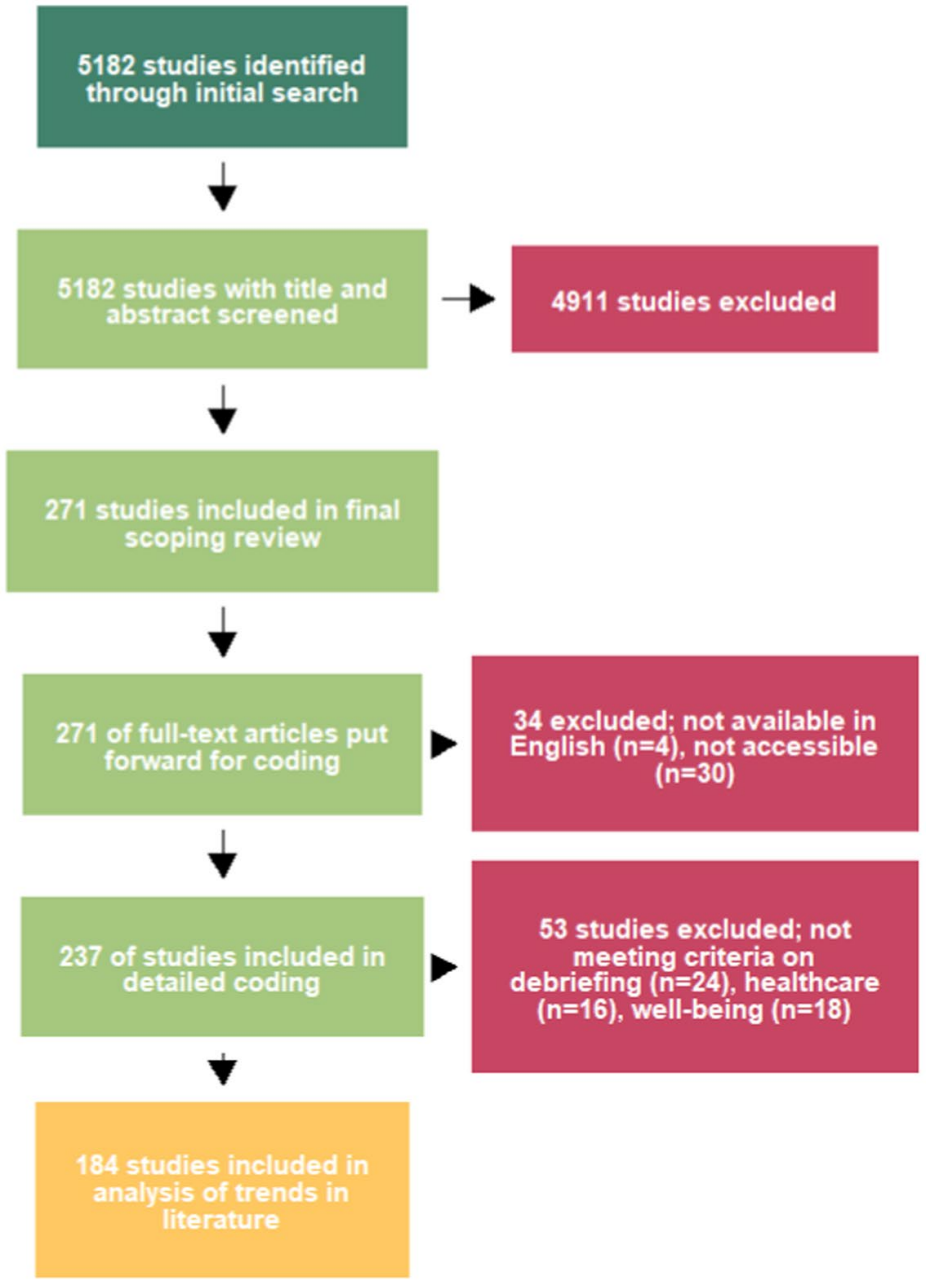
PRISMA-inspired flow diagram.

In total, 184 papers were deemed to meet the three inclusion criteria and were fully coded. We used R Core Team (32) and RStudio Team (33) for data management and analyses, including the dplyr (34) and readr (35) packages. See Figure 2, produced using metagear (36). The coded data were then combined with the bibliographic data collected through the original literature review, and can be found on the OSF project page (see footnote 4).

## Results

### Study characteristics

In sum, 184 studies are included in the scoping review and were deemed suitable for analysis of the trends in the literature. See^6^ outlining each study and accompanying coding. As this is a mapping/scoping review, we first present a publishing timeline (see Figure 1), indicating that numbers of publications in the area have increased rapidly in the last decade.

Second, we identified the locations and journals which have more commonly disseminated such works. Twenty-one locations were represented, yet work was predominantly conducted within the US (*n* = 67), Australia (*n* = 29), the UK (*n* = 26) and Canada (*n* = 21). The places of publication were more widely distributed, with 140 publication destinations identified. The journals identified mostly featured one or two publications, with the exceptions of Clinical Simulation in Nursing (*n* = 7), Nurse Education in Practice (*n* = 6), Journal of Clinical Nursing (*n* = 5), Prehospital & Disaster Medicine (*n* = 5), Emergency Medicine Australasia (*n* = 4), Emergency Medicine Journal (*n* = 3) and the International Journal of Environmental Research and Public Health (*n* = 3). Co-authorship mapping was also conducted but indicated negligible collaboration between researchers in the field.

Third, with respect to design, the works featured were predominantly empirical (*n* = 119), but also included editorial/opinion papers (*n* = 27), reviews (*n* = 23), case studies (*n* = 14) and a study protocol (*n* = 1). Research typically focused upon debriefing following real-world events (*n* = 159), although there was an emerging body of work using simulation (*n* = 22) and three papers had elements of both simulation and real-world settings.

**Figure 3:**
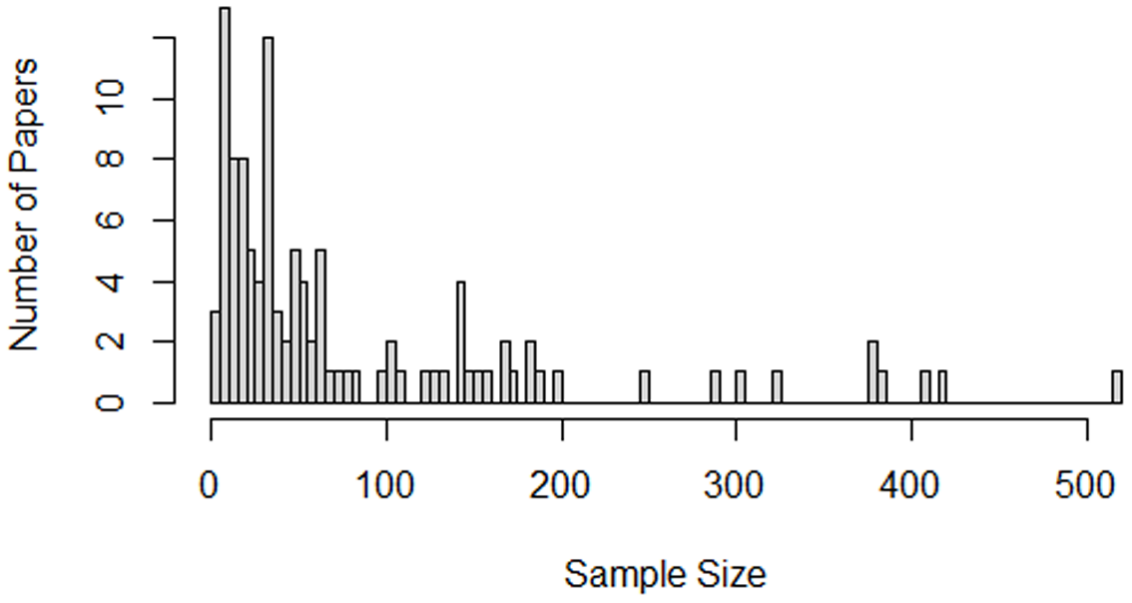
Sample size of manuscripts following exclusion of 3 (*N* > 1,000) studies.

Fourth, empirical works with a coded sample size (*n* = 112) represented between 3 and 3,822 participants (*M* = 141.63). Three papers had more than 1,000 participants, and after excluding these papers the mean sample size decreased meaningfully (*n* = 109, *M* = 83.54). The sample size distribution is heavily positively skewed (see Figure 3), with 79 of the 112 papers representing less than 100 participants. While the papers in this category represent both qualitative and quantitative works, which may contribute to this trend for small sample sizes, it should be noted that the largest sample size (*n* = 3,822) was from a qualitative study (37).

### Keyword mapping

**Figure 4:**
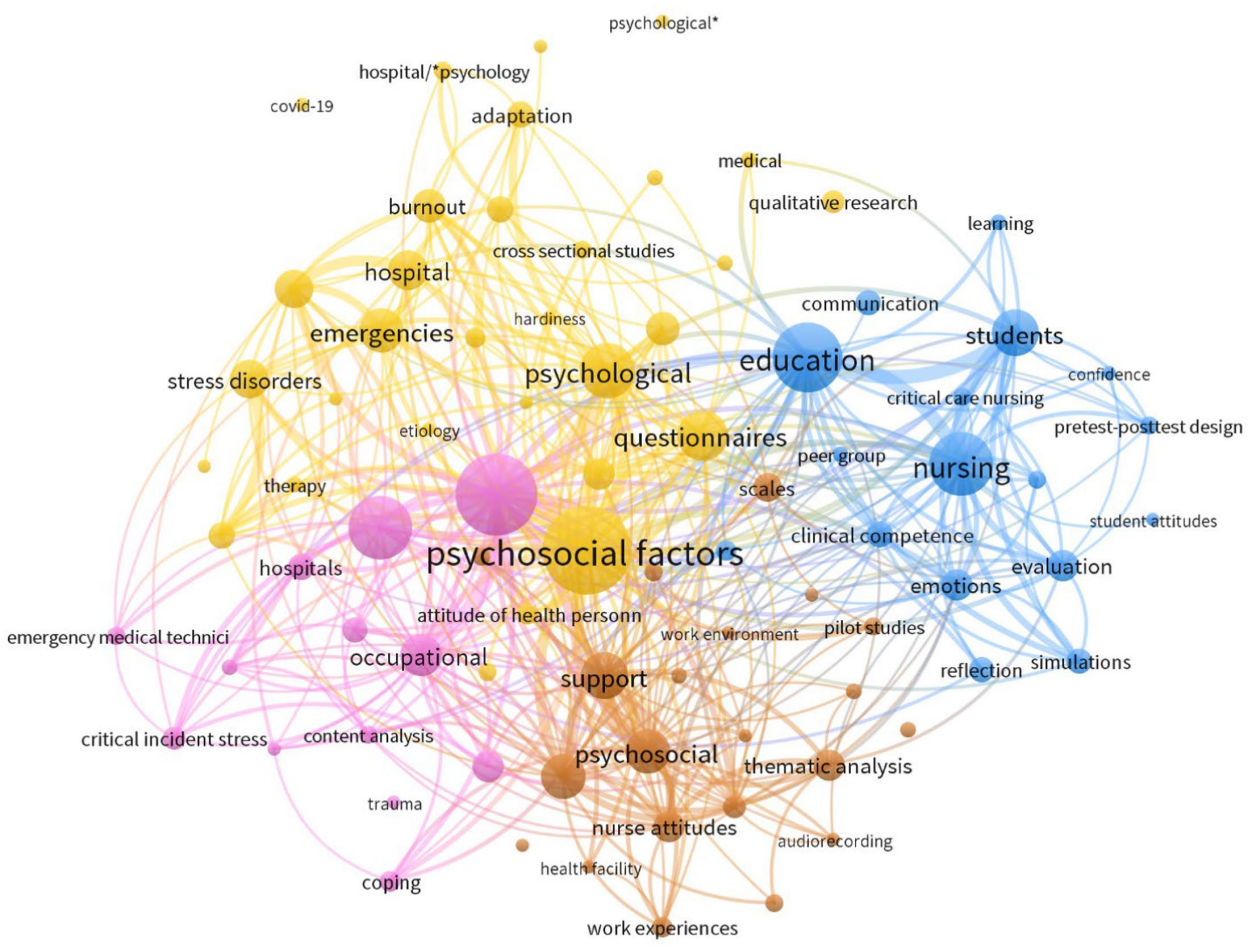
Keyword mapping.

We explored the mapping of keywords and explored any meaningful groups or subgroups of literature using VOSviewer (38). A co-occurrence analysis of 839 keywords was carried out. The minimum occurrence of a keyword was set to 5, which left 100 words that met the threshold. In this analysis, we manually removed terms relating to procedure (descriptive statistics, data analysis software, funding source, methods, convenience sample, *t*-tests, summated rating scaling) and sample (human, female, male, adult, middle age, humans, purposive sample, Australia, middle aged, infant, and newborn). The method of normalization was association strength. Following an iterative and exploratory clustering process, the minimum cluster size was set to 12 and in total, 4 clusters were produced with 1731 links. This mapping, as seen in Figure 4, is roughly indicative of a number of bodies of literature. Prevalence is indicated by circle size and co-occurrence is demonstrated through width of connecting lines. The words in yellow focus upon psychological states and represent the most common research types (e.g. cross-sectional studies) adopted within the literature. Words in blue focus more on the practical literature with student samples, simulations and that conducted for educational purposes. The brown words are indicative of the research surrounding support for staff and the mostly qualitative research exploring workplace experiences. Finally, pink represented the literature on critical incidents, stress and coping of staff. This mapping was created in a highly exploratory style and should not be seen as an approach to grouping bodies of research. Nevertheless, it does provide an initial view of how certain themes co-occur across the existing literature and where they may be opportunities to unify and create consensus.

### Exploratory analyses

Finally, we explored trends within the literature based upon the various extraction criteria to identify areas of strength from which practice recommendations could be made more confidently, and highlight areas of potential development.

When discussing the purpose of the debrief, 102 papers captured debriefing in response to a specific incident or event, while 77 considered routine debriefing, and 5 papers captured elements of both. Populations discussed commonly focused upon nursing (*n* = 93) and working in emergencies (e.g. emergency departments; *n* = 22), with many works discussing specific populations, e.g. doctors (*n* = 17) and students (*n* = 34), and some covering a range of disciplines or population types (*n* = 25).

With respect to models or approaches to debriefing, 65 papers stated no clear strategy, 28 noted critical incident stress debriefing, 13 noted critical incident stress management and 89 noted a diverse range of strategies including ‘hot’ debriefing, peer/group support, huddles, Schwartz rounds and bespoke models, tools and approaches. With respect to facilitation, nearly half of the studies did not report the individual responsible for the debrief (*n* = 91). Using internal staff (e.g. senior staff or colleagues; *n* = 50) or the involvement of the researcher/educator (*n* = 18) was much more common than external facilitation (*n* = 7). Nineteen studies made reference to facilitators but were ambiguous as to their role.

Whether perceived or measured, a diverse range of emotional outcomes were claimed from debriefing, most commonly including stress (*n* = 42), post-traumatic stress disorder symptomatology (*n* = 36), wellbeing (*n* = 25), burnout (*n* = 21), and coping (*n* = 15). Other popular outcomes included specific emotional experiences like grief and anxiety, and more general terms including resilience, moral distress, compassion fatigue and emotional support.

A range of exploratory analyses (predominantly Chi-squared tests) were conducted to identify whether certain combinations of the coded features occurred more frequently, thereby representing trends within the literature. For example, relative to real-world work, simulation studies were less common in Australia (1/29) and the UK (0/26), compared to South Korea (4/5), Canada (3/21) and US (6/67). The majority of these exploratory analyses were non-significant and/or based upon insufficient numbers and were therefore mostly inconclusive. Together with the analyses presented above, the results are indicative of sporadic research attention.

## Discussion

Following a rigorous review process, we coded 184 relevant academic sources of evidence considering the role of debriefing on the wellbeing and emotional outcomes of healthcare staff. Little of the work provided empirical evidence of how to negotiate employee voice for the employee’s affective benefit. We can conclude that the body of evidence is clustered geographically, but diverse on many other criteria of interest including the types of evidence produced, debriefing models and practices and outcomes captured. This diversity, likely manifesting the desire for novelty in research, should be considered problematic for building incremental understanding and convincing bodies of evidence from which practice can be informed. Efforts to evaluate local practice were common, but with limited efforts to incrementally build understanding and draw a consensus (e.g. in specific debriefing practices or measurement of outcomes), there are many barriers to confidence in subsequent conclusions.

Given the importance of healthcare workers and their voice, the state of current evidence is concerning and at risk of being considered too disparate for informing practice. The majority of studies in the field have been published since 2015 and while there does appear to be a dramatic increase in research published on this theme, this trend loosely maps onto the observed increase in research outputs in most healthcare and psychology domains over this same time period (39) and is indicative of limited prioritization of this field. Similarly, the co-author mapping was indicative of a highly siloed approach to working, where there were little-to-no links between different publication teams. Given the extent of diversity in methods and approaches available for exploring the impact of debriefing, this field would benefit from greater collaborative initiatives to collect evidence where consensus could help inform practice. Use of adversarial collaborations to cohere these diverse bodies of evidence [e.g. (40)], or increased transparency initiatives to better share resources, materials and data, would appear to be of particular benefit. The keyword mapping in Figure 3 tentatively provides a number of areas which may help unify this disparate landscape (e.g. considering both individual, social and contextual factors), and areas where clear gaps exist (e.g. debriefing for critical incidents in student populations).

The sporadic nature of the literature identified makes it clear that there are not standardized approaches, tools or practices to impact and assess the wellbeing and emotional outcomes of healthcare workers’ debriefs. This conclusion is similar to that previously noted by Richins et al. (18). While many shared terms and principles are adopted, there are limited similarities in practice which would support synthesis of conclusions. Furthermore, there is a clear lack of focus on the mechanisms by which such practices could give healthcare workers voice and impact specific emotional outcomes. For example, while some specific practices were considered, e.g. use of a moment of silence (41), little work focused on the nuances of when, how and why they should use their voice or work with the silence. To ensure this body of evidence can support healthcare workers, research in this field should prioritize areas for unification, including development of a comprehensive model, standards for debriefing practice, systematic reporting guidelines and further structured evidence mapping/syntheses. Researchers attempting further empirical work are encouraged to collaborate to build more rigorous bodies of evidence on specific debriefing types, and to increase the transparency of dissemination, to offer the greatest likelihood of informing consensuses for practice.

Any further conclusions drawn from this work should be made in context of our scope and limitations. For example, our review was conducted using Western databases and consisted of studies adopting the English language. We also deviated from our preregistration to provide a clearer statement on the current state of academic understanding, at the cost of omitting a number of crucial non-academic evidence sources like professional practice guidelines. We therefore conclude our findings represent only part of a much wider body of evidence in the field. There are plenty of highly informative expert and experience-based evidence sources in this field (42). Furthermore, while our coding process required multiple trained reviewers and the estimates of inter-rater reliability were promising, the definition of our coding criteria were *ad-hoc* and required subjective decision-making that may compromise the representation of the evidence sources. We encourage researchers interested in conducting further reviews of the field to focus on specific debriefing practices or outcomes, using the current review resources for search validation, and to expand their review scope to consider the full range of grey literature and non-academic sources that could inform practice.

The current review excluded a large number of sources based upon their inaccessibility to the research team. As we are a relatively privileged group, holding institutional subscriptions and access to a number of additional approaches to read otherwise inaccessible work, this should be considered highly problematic. Furthermore, we saw little evidence of transparency, with few papers sharing detailed information on debriefing, only one paper representing a preregistration and little-to-no data sharing. We believe this is reflective of the wider body of evidence, and we encourage researchers in this domain to prioritize accessibility and transparency through open scholarship practices like preprinting, open materials and data and sample size planning.

Finally, the current review did not evaluate the evidence for the efficacy of debriefing. Recommendations for practice should only be made upon the basis of a critical evaluation of all existing evidence sources, and this was not possible from the current review. When coding the work, a number of claims for both positive and negative outcomes were identified, and the debriefing practices, size of effects and outcomes captured tended to be highly diverse. For example, differences in the valence of outcomes were anecdotally observed between routine debriefing and strategies for managing traumatic experiences. Having coded much of the academic literature, we hope the resources and work produced here provides a springboard to complete this important work evaluating debriefing efficacy, and other such works considering important factors like profession or debriefing model. Furthermore, given the extent of case studies, editorial/opinion claims and generally low sample sizes identified, we hope this evidence review will place great emphasis upon reviewing evidence quality. To contribute toward these goals in advancing evidence and practice, subsequent work in this field should avoid making strong claims about debriefing efficacy and prioritize generating quality evidence with transparency trails to facilitate effective evaluation and synthesis.

## Conclusion

The emotional wellbeing of healthcare staff has been neglected and under-acknowledged, leading to substantive numbers of staff experiencing moral injury, stress and burnout (43). Debriefings are likely to give healthcare employees voice, to not only improve clinical outcomes but to improve their own emotional experiences and wellbeing at work (44). The results of our systematic scoping review about the evidence on debriefing practices for the wellbeing and emotional outcomes of healthcare workers are indicative of a fragmented and inconclusive body of literature on this topic. Structured, collaborative and transparent approaches to expand the theoretical and empirical evidence available to inform practice are vital to learn how best to give healthcare employees voice, and to promote better wellbeing and emotional outcomes.

## Author contributions

TE, RE, CB, GF, and SW: conceptualization and methodology. TE and RE: software, analysis, visualization, and project administration. TE, RE, CB, and GF: validation. AC, BH, BM, EH, FQ, GB, GW, HE, JC, JM, JS, KJ, LS, LJ, MC, MD, MM, PC, PG, WM, and YY: investigation. TE: data processing and supervision. CB, EH, GF, and TE: writing–original draft. AC, BH, BM, CB, DM, EH, FQ, GB, GF, GW, HE, JC, JM, JS, KJ, LJ, LS, MC, MD, MM, PC, PG, RE, SW, TE, WM, and YY: writing–review and editing. All authors contributed to the article and approved the submitted version.

## Conflict of interest

The authors declare that the research was conducted in the absence of any commercial or financial relationships that could be construed as a potential conflict of interest.

## Publisher’s note

All claims expressed in this article are solely those of the authors and do not necessarily represent those of their affiliated organizations, or those of the publisher, the editors and the reviewers. Any product that may be evaluated in this article, or claim that may be made by its manufacturer, is not guaranteed or endorsed by the publisher.

## Footnotes

^1^ https://osf.io/za6rj

^2^ https://osf.io/h5ezk

^3^ https://www.youtube.com/watch?v=xPxzmtH1oTw

^4^ https://osf.io/6fh39/

^6^ https://osf.io/qdfk8

## Appendix 1: Full search criteria

Debrief* OR Prebrief* OR briefing OR huddle

AND

Doctor OR physician OR clinician OR “medical practitioner” OR nurs* OR “health profession*” OR healthcare OR “health care” OR pharmac* OR dentist OR midwi* OR dieti* OR “occupational therap*” OR paramed* OR physiotherap* OR radiograph* OR psycholog* OR “health worker” OR hospital

AND

Emotion* OR feeling* OR wellbeing OR wellbeing OR cope OR coping OR mood OR affect* OR positiv* OR negativ* OR stress* OR wellness OR resilien* OR “mental health” OR anxiety OR depress* OR burnout

## Appendix 2: Contributorship

The current project adopted the CRediT taxonomy by which we acknowledge the contributions of all individuals who worked on the current project.

**Table 1:**

Term	Definition	Contributors
Conceptualisation	Ideas; formulation or evolution of overarching research goals and aims	Project development: TRE; RE; CB; GF; SW
Methodology	Development or design of methodology; creation of models	Study design: TRE; RE; CB; GF; SW
Software	Programming, software development; designing computer programs; implementation of the computer code and supporting algorithms; testing of existing code components	Rayyan, VOSViewer, etc.: TRE & RE
Validation	Verification, whether as a part of the activity or separate, of the overall replication/reproducibility of results/experiments and other research outputs	Third coding: TRE; RE; CB; GF
Formal analysis	Application of statistical, mathematical, computational, or other formal techniques to analyze or synthesize study data	Analyses: TRE & RE
Investigation	Conducting a research and investigation process, specifically performing the experiments, or data/evidence collection	First/Second coding: AJC; BH; BM; EH; FQ; GB; GVW; HE; JC; JMT; JS; KJ; LDS; LJ; MAC; MLD; MM; PC; PG; WM; YY
Resources	Provision of study materials, reagents, materials, patients, laboratory samples, animals, instrumentation, computing resources, or other analysis tools	N/A
Data curation	Management activities to annotate (produce metadata), scrub data and maintain research data (including software code, where it is necessary for interpreting the data itself) for initial use and later reuse	Data processing: TRE
Writing–original draft	Preparation, creation and/or presentation of the published work, specifically writing the initial draft (including substantive translation)	Involved in writing: CB; EH; GF; TRE
Writing–review & editing	Preparation, creation and/or presentation of the published work by those from the original research group, specifically critical review, commentary or revision—including pre- or postpublication stages	Involved in editing: All authors
Visualization	Preparation, creation and/or presentation of the published work, specifically visualizsation/data presentation	Creating visualization: TRE & RE
Supervision	Oversight and leadership responsibility for the research activity planning and execution, including mentorship external to the core team	Supervision: TRE
Project administration	Management and coordination responsibility for the research activity planning and execution	Project admin: TRE & RE
Funding acquisition	Acquisition of the financial support for the project leading to this publication.	Funding: CB
